# Smart Corrosion Monitoring in AA2055 Using Hidden Markov Models and Electrochemical Noise Signal Processing

**DOI:** 10.3390/ma18122865

**Published:** 2025-06-17

**Authors:** Cynthia Martinez-Ramos, Citlalli Gaona-Tiburcio, Francisco Estupiñan-López, Jose Cabral-Miramontes, Erick Maldonado-Bandala, Demetrio Nieves-Mendoza, Miguel Angel Baltazar-Zamora, Laura Landa-Ruiz, Ricardo Galvan-Martinez, Facundo Almeraya-Calderón

**Affiliations:** 1 Universidad Autónoma de Nuevo León Centro de Investigación e Innovación en Ingeniería Aeronáutica (CIIIA), FIME, San Nicolás de los Garza 66455, Mexico; cynthia.martinezrms@uanl.edu.mx (C.M.-R.); citlalli.gaonatbr@uanl.edu.mx (C.G.-T.); francisco.estupinanlp@uanl.edu.mx (F.E.-L.); jose.cabralmr@uanl.edu.mx (J.C.-M.); 2 Facultad de Ingeniería Civil, Universidad Veracruzana, Xalapa 91000, Mexico; erimaldonado@uv.mx (E.M.-B.); mbaltazar@uv.mx (M.A.B.-Z.); lalanda@uv.mx (L.L.-R.); 3 Instituto de Ingeniería, Universidad Veracruzana, Calz Juan Pablo II S/N, Fracc. Costa Verde, Veracruz 94294, Mexico; rigalvan@uv.mx

**Keywords:** corrosion, electrochemical noise, passivation, models, aluminum

## Abstract

This work explores the application of Hidden Markov Models (HMMs) for the classification and reconstruction of corrosion mechanisms in the aerospace-grade aluminum alloy AA2055 from the signals obtained by electrochemical noise (EN) analysis. Using the PELT algorithm to segment the signal based on relevant changepoints, distinct corrosion states within the segments are isolated and identified, including general, localized, and mixed corrosion based on statistical signal features, which are used to create the probabilistic structure of HMMs through the initiation, transition, and emission matrices. This study utilized a dataset composed of five electrolyte groups, each containing ten EN signals with 1024 data points per signal, totaling 51,200 data points. The model demonstrates that even with variability in signal quality, meaningful reconstruction is achievable, especially when datasets include distinct transient behavior.

## 1. Introduction

Effective maintenance planning for industrial sites relies on factors such as equipment age, operational environmental conditions, and human involvement [1,2,3,4], all of which will ultimately have a direct or indirect involvement in its service life. A critical part of safeguarding and extending the durability of the components and machinery comes down to prediction and life expectancy projections [4,5,6].

With corrosion at the forefront of our research, identifying the most suitable evaluation technique for a comprehensive understanding of the process is required. Electrochemical techniques stand out from other approaches as they offer significant advantages: they enable the real-time in situ monitoring of corrosion processes under realistic conditions. Additionally, most electrochemical tests are non-destructive, allowing for repeated measurements without compromising the integrity of the sample. Despite these advantages, a point of contingency is that often, electrochemical methods require careful interpretation, as results can be influenced by multiple factors, and if they are not properly considered, they can lead to misinterpretations [7,8]. Electrochemical noise allows the analysis of the test subject’s real response to the exposure to a given electrolyte, with no external perturbation introduced to the system being analyzed under open-circuit conditions. The sensitivity of the fluctuations gives access to valuable information about the present process, such as the dissolution, passivation, and corrosion mechanisms, through the behavior, patterns, and signatures shown in the signal [7,8,9,10]. Interpretation can be approached through direct examination, statistical or frequency-based methods, or a combination of both.

Among the most common approaches for analyzing electrochemical noise (EN) signals is the visual inspection of time domain patterns, which depends heavily on the researcher’s experience and may lack precision and reproducibility [10,11,12,13]. Statistical analysis of EN involves evaluating how data points deviate from the mean, often under the assumption of a Gaussian distribution, to infer corrosion mechanisms [13]. While this simplification facilitates modeling, it may not be suitable for all signals, particularly those that deviate significantly from normality. For improved accuracy, preliminary distribution fitting is recommended before applying Gaussian-based metrics. Closely related to this, signals with little to no noticeable variability will introduce uncertainty in high-order statistical parameters. Additionally, values such as the localization index or coefficient of variation have fallen out of use because they become problematic when the mean approaches 0, as they lead to undefined or misleading results and are only valid under one-sided distributions [10,11,12,13,14].

Frequency-based analysis has become a growing niche on its own merit, transforming the time-based data into a power spectrum that enables the calculation of metrics such as noise impedance. The versatility provided by the power spectral density has been proven by many authors, whose works further elucidate the applications of techniques such as wavelets, chaos theory, and fractals, amongst many others, which could not otherwise be performed under a different approach [12,13,14,15,16,17,18]. However, this approach demands a strong theoretical background and involves longer processing times compared to statistical techniques. A particularity of the frequency-based approach is its lack of an intrinsic classificatory metric. Rather than producing a numerical threshold or index for corrosion types, it relies on expert interpretation of spectral features such as the PSD slope, whose diagnostic value remains context-dependent and without consensus. Features such as the Lyapunov exponent have been proposed to differentiate corrosion mechanisms based on system chaos [19,20,21,22]. Nevertheless, these and other frequency-based calculations have yet to be standardized as they remain in the experimental phase. The caveat of this technique is that, due to its complexity, it is often better suited for academic research and not for on-site monitoring [13].

Having explored both the time and frequency domains, keeping in mind the current demand for rapid, reliable, and non-destructive monitoring techniques, this research aims to develop a tool that enhances corrosion monitoring in situ, improving the robustness and efficiency of current techniques. Statistical analysis in the time domain is selected for its long-standing use and its classificatory nature, which is essential for understanding corrosion during monitoring. This domain also complements visual inspection, which, together with statistical methods, remains a mainstay of in situ monitoring. Despite their strengths, little innovation has occurred in this area.

To address these limitations, this work integrates signal segmentation using an adapted Pruned Exact Linear Time (PELT) method. This approach isolates regions of interest within the signal, replacing interpretative detection of shifts with a numerically sound framework. Statistical features are then extracted from each segment, reducing the influence of the central limit theorem, which often weakens accuracy. This also allows for a more detailed view of how corrosion evolves over time, beyond just dominant trends.

Finally, these features will serve as inputs for a predictive model based on Hidden Markov Modeling (HMM), allowing the framework to detect evolving trends and anticipate future corrosion behavior. HMM uses classificatory features (in this case, corrosion types) to predict which type is most likely to follow, based only on the current state. To do this, an initiation matrix and a transition matrix will be built to represent the probabilities of starting in and shifting between corrosion types. An emission matrix will also be created to indicate the likelihood of a data point belonging to each type. These matrices will be used to reconstruct the signal path, identifying when different corrosion types are likely to occur.

The main purpose of this study is to develop a prediction-based algorithm tailored to EN signals that can be applied to various metallic materials under different corrosive environments, with the intent of improving the reliability and depth of corrosion classification beyond what traditional techniques can offer. To achieve this, it is important to successfully implement PELT segmentation prior to HMM to provide the necessary classificatory features required for the probability calculations and, additionally, overcome the identification of the most suitable penalization value without arbitrary values.

The introduction of these methodologies diverges from the current works and offers a new insight through the introduction of Markovian principles into electrochemical noise analysis in an optimized environment that streamlines automatic segment detection, statistical classification of corrosion mechanisms, and subsequent Markov reconstruction of the signals. While previous studies have centered mostly on either visual inspection, traditional statistical parameters, or advanced frequency domain techniques, to the authors’ knowledge, no previous framework has combined numerical segmentation with an HMM-based probabilistic model to capture the dynamic evolution of corrosion for in situ corrosion monitoring, making this a novel contribution.

### Theoretical Framework

In the context of electrochemical noise, the earliest documented usage of statistical parameters occurred during the late 1990s [23,24,25,26,27,28], making it a long-known technique but not widely adopted by many. At the time of writing, a Google Scholar search for the term “electrochemical noise corrosion” revealed 44 publications between the decade of 1990 and 2000; this number increased considerably (to 198) in the following decade (2000–2010), which rose further to 446 during 2010–2020 and reached a total of 554 works in the most recent five-year period (2020–2025). This goes to show that there has been a notable increase in the scientific community’s interest in the implementation of EN and the interpretation of its results. Still, the lack of its widespread use could be derived from the sensitivity of the technique to environmental factors, drift, as well as the processing time and skillset required to interpret the signal’s response, with alternatives like electrochemical impedance spectroscopy (EIS) or linear polarization resistance (LPR) offering more straightforward results and established interpretation protocols, albeit with their own inherent limitations. In spite of this, electrochemical noise has the capability of capturing the spontaneous natural fluctuations in current and potential, with no external perturbation introduced; this feature means that underneath the data we gather, we obtain the true real-time response, with no accelerations in the system or adverse interventions [29]. This is what, in turn, makes the arduous process of unearthing what lies hidden within the signals and determining why, in spite of its drawbacks, EN has been rising since its introduction all the more meaningful.

In a study, Homborg et al. [30] used the time–frequency domain, with the aim of identifying pitting corrosion through image recognition machine learning in the form of a convolutional neural network of transients to reduce human interpretation by the automatic verification of specific local frequency information within a transient. Bongiorno et al. [31] developed a model for the detection of passivity and localized and general corrosion; using AA 6082 T3, pure aluminum, carbon steel, and 310 SS, the authors calculated statistical parameters to feed into a random forest model (RFM). *Calabrese* et al. [32] performed data mining in the time–frequency domain through the Hilbert–Huang transform (HHT) to identify stress corrosion cracking by decomposing electrochemical noise signals into intrinsic mode functions, extracting statistical descriptors, and applying a data mining algorithm to classify damage stages. Other works [33,34,35] have also shown interest in the pursuit of novel algorithms, machine learning, and artificial intelligence as a means to support electrochemical noise evaluation.

In order to support a dynamic and probabilistic analysis of electrochemical noise signals, two methods are introduced and used in tandem: Pruned Exact Linear Time (PELT) and Markov modeling. First, to segment the signal into meaningful sections by identifying the changepoint in the data, statistical features required for categorization are extracted, after which Hidden Markov Modeling (HMM) is used to generate a predictive model for corrosion progression by using the statistical descriptors in the previous step. It is worth noting that PELT functions as a standalone method in and of itself and could be used for other purposes, while MM relies on categorized input data to source its predictions, not necessarily obtained from PELT. The combination of these two approaches in the context of EN, to the best of the authors’ knowledge, represents a novel integration, with the aim of automating the identification of areas of interest within the signal from a mathematical perspective and generating a predictive model that does not rely on a large database. Instead, it leverages the Markovian property of predicting future states based solely on the present one after learning from the statistical characteristics extracted from the data.

- *Pruned Exact Linear Time Algorithm*

Over the years, many changepoint detection algorithms have been introduced, many of which have a considerable computational cost, hindering the processing time and the quantity of data being able to be analyzed in a single run. The PELT method was introduced by Killick [36] as an optimization of the Optimal Partition method [37], in which the data $y=\left\{y_{1},\;y_{2},\;\ldots y_{n}\right\}$ are segmented into *m* segments, whose changepoints are located at $\tau_{1},\tau_{2},\;\ldots \tau_{n}$. These points are determined by minimizing the total cost, defined as the sum of the fitting cost for each segment plus a penalty term β that controls the number of segments and does not depend on the number or location of changepoints. The configuration that yields the lowest total cost identifies the optimal changepoints, i.e., *F*.

It is important to note that in this context, the term “cost” refers to a measure quantifying how well a given segment fits the data, and its calculation can be performed using different models depending on the behavior of the data [38]. The modification introduced by Killick includes a pruning step to improve the computational efficiency without sacrificing the calculation of the global minimum cost. This is achieved by discarding changepoint candidates *τ*, which can never be part of an optimal segmentation, based on prior cost evaluations, guaranteeing an exact globally optimal solution under a specified cost model and additive penalty structure (see Equation (1)):

(1) $$F\left(n\right)=\begin{array}{c} min\\ \tau \end{array}\left\{\sum_{i=1}^{m+1}\left[C\left(y_{\tau_{i-1}+1},\ldots ,y_{\tau i}\right)+\beta \right]\right\}$$

- *Hidden Markov Models*

A Hidden Markov Model describes a system in which the observation of interest is produced by, or is closely dependent on, a hidden process, within which the data that describe our observations are located. The founding pillars of this technique are the Markov chains, which describe a stochastic process or sequence in which the future state does not depend on the past, only on the present. Its creation is attributed to the Russian mathematician Andrey Markov, who developed the algorithm in order to demonstrate that the two fundamental theorems of probability—the weak law of large numbers and the central limit theorem—could be extended to sums of randomly dependent variables through the theory of determinants of finite square matrices [39].

The property of Markov chains is best represented by Equation (2):

(2) $$P(q_{i}=a|q_{1}\;\ldots \;q_{i-1}\;)=P(q_{i}=a|q_{i-1})$$

where every state is represented by variables of aleatory states $Q=q_{1},\;q_{2},\;\ldots ,\;q_{i}$ that evolve around a sequence, indicating that the probability of transition to a new state *q_i_* depends only on the current state and that no prior states are involved [40].

State transitions are quantified by means of a transition probability matrix $A=\left[a_{ij}\right],$ where each element *a_ij_* represents the probability of moving from state *i* to state *j*, and all possible present states must be considered. Additionally, all values must be positive, and the sum of each row must always equal 1 [40,41]; this is part of Markov’s theory as all states considered are expected to occur at some point in their own capacities; thus, having a negative or a null value would not be possible. The next component consists of the initial state probability distribution, which represents the probability that the Markov chain begins in a given state (*i*): $\pi =\pi_{1},\;\pi_{2},\ldots \pi_{N}$. This value is known or can be obtained by analyzing the first state of the dataset itself to determine an average.

Having established the basis for HMM, the implementation of this technique centers around a sequence of events where the states of interest cannot be directly observed, hence the term ‘hidden’. This model represents the probability distributions over a sequence of observations (*O*) at time *t* produced by a stochastic process, where the state of interest (*Q*) is hidden; the state transitions are of a probabilistic nature, and the observations are probabilistic functions of the state. In a first-order HMM, two key assumptions are made (see Equation (3)) [41,42]:

(3) $$P\left(q_{i}\left|q_{1}\ldots q_{i-1}\right.\right)=P(q_{i}\left|q_{i-1}\right.)$$

The second assumption states that the probability of an output observation *oᵢ* at time *t* depends only on the current state *qᵢ* that produced the observation, not on any other state or any other observation. This can be written as Equation (4):

(4) $$P\left(O_{i}\left|q_{1}\ldots q_{i}\ldots q_{T},O_{1},\ldots ,O_{i},\ldots ,O_{T}\right.\right)=P(O_{i}\left|q_{i}\right.)$$

The two assumptions lead us to the following expression, which represents the joint probability of a sequence of observations O_1_:N and states Q_1_:N (see Equation (5)) [43]:

(5) $$P\left(O_{1:N},Q_{1:N}\right)=P(Q_{1})\prod_{t=2}^{N}P(Q_{t}\left|Q_{t-1}\right.)\prod_{t=1}^{N}P(O_{t}\left|Q_{t}\right.)$$

The relevant probability features required to perform HMM are as follows [42,44,45]:

The probability distribution of transitioning between states (*B*) is shown in Equation (6).

(6) $$a_{ij}=P\left[q_{t+1}=q_{j}|q_{t}=q_{i}\right],\;1\leq i,\;j\leq N$$

Emission probability, which describes the probability that a given observation will be produced by a certain state, is shown in Equation (7).

(7) $$B=bi(o_{t})=P\left[o_{t}\;|q_{t}=q_{j}\right],\;1\leq j\leq N$$

For robust and correct use of the model, the three prompts introduced by Rabiner must be addressed and solved [45]. The first prompt ensures the correct evaluation of the probability calculation of the sequence of observations. The second one encompasses the decodification step, which defines the choice of state sequence that best fits the observations. The third one emphasizes the learning feature of the algorithm and how the parameters of the model must be adjusted through the Baum–Welch algorithm. The specifications of the algorithms mentioned and their nuances fall outside the scope of the present research; thus, readers are directed to [46,47,48] for further clarification.

## 2. Materials and Methods

### 2.1. Materials

The material selected for this study was an AA 2055 aluminum–lithium alloy in an as-received condition, with no external coating, painting, or heat treatment. The AA2055 was analyzed by X-ray fluorescence (Olympus DELTA XRF., Richmond, TX, USA) to obtain its chemical composition (see Table 1).

The aluminum samples were mechanically polished using metallographic techniques according to the ASTM E3-11 standard [49]. The material was sequentially polished using different SiC grit papers with 400, 600, 800, and 1200 grades. The samples were rectangular coupons measuring approximately 2.5 cm × 5 cm. During testing, only a circular area of 1 cm in diameter was exposed to the electrolyte through the cell opening, while the rest of the sample’s surface was isolated. To ensure a dataset with significant variability, five electrolytes were selected: 3.5 wt.% NaCl, 5 wt.% H_2_SO_4_, and 5 wt.% HCl, each prepared using deionized water, along with tap water samples from Monterrey, Nuevo León, and Gómez Palacio, Durango, Mexico. The selection process for these electrolytes required obtaining a varied response of the samples to different environments to gain a well-rounded database to work with. The 3.5 wt.% NaCl solution simulates a marine or saline environment, which is frequently used as a standard for corrosion testing due to its relevance in coastal or offshore industrial settings. The 5 wt.% H_2_SO_4_ and 5 wt.% HCl solutions are representative of acidic industrial environments with aggressive corrosion mechanisms, where sulfuric and hydrochloric acids are commonly used in processes such as pickling, acid cleaning, or chemical manufacturing [50,51,52]. Lastly, the tap water samples were included to simulate realistic and uncontrolled exposure conditions, introducing variability from natural ionic content, hardness, and potential microbial activity.

**Figure 1:**
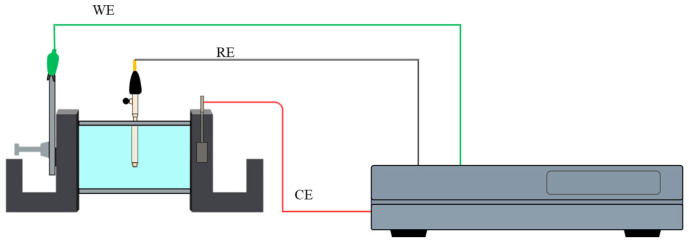
Electrochemical noise testing for the conventional three-electrode cell system [21].

Electrochemical noise (EN) testing was carried out at room temperature following the ASTM G199-09 and ISO 17093:2015 standards [22,23,24,25], using a VersaSTAT3 potentiostat/galvanostat/ZRA (Zero Resistance Ammeter) (AMETEK, TN, USA). The setup consisted of a conventional three-electrode cell, configured symmetrically: the samples served as the working electrode (WE), a saturated calomel electrode (SCE) was used as the reference electrode (RE), and a platinum mesh functioned as the counter electrode (CE), as shown in Figure 1. Electrochemical noise experiments were performed 10 times for each electrolyte. EN data were recorded at a sample rate of 1 point per second, for a total of 1024 data points per test.

All calculations were performed in *Python* programming language version 3.13.1 and its associated libraries, using *Visual Studio Code* (version 1.101) as the integrated development environment (IDE). Prior to analysis, all signals underwent preprocessing using a 9th-degree polynomial filter to remove parasitic currents and extreme outliers [53,54].

### 2.2. Signal Segmentation and Classification

The PELT algorithm from the ruptures library was used to identify meaningful changepoints. The model selected for this task was radial basis function (RBF) [55] to obtain the cost function in the form of a kernel by calculating the Euclidean distances between the data points with respect to a reference centroid within the dimensional space (see Equation (8)).

(*x*, *y*) = exp(−γ ‖ x − y ‖)^2^ (8)

The penalty value has a major influence on the quantity of generated segments and detection sensitivity. A sensitivity assessment was performed over a range of 4 to 40 possible changepoints. The optimal penalty value was determined automatically by iterating through the segmentation outcomes and selecting the value that yielded the most consistent and stable results. The minimum number of data points per segment was established at 30 data points. This value was selected in accordance with Reid [56], who stipulated that the data range for calculating kurtosis and skewness in corrosion research must be within 30 to 2048 data points. After successful segmentation by the changepoints, SD, kurtosis, and skewness were calculated for all segments generated, and the results were categorized by general or localized corrosion in accordance with Reid’s proposed values (see Table 2).

### 2.3. Signal Segmentation and Classification

Skewness is a third-order statistical moment that measures the asymmetry of a probability distribution relative to its mean. Positive skewness typically reflects anodic transients, while negative skewness is associated with cathodic events: see Equation (9) [57,58].

(9) $$s=\frac{\frac{1}{N}\sum_{i=1}^{N}{\left(x_{i}-\bar{x}\right)}^{3}}{\sigma^{3}}$$

Kurtosis corresponds to a fourth-order statistical moment that characterizes the sharpness of the peak and the heaviness of the tails of a probability distribution. Leptokurtic values (kurtosis > 3) suggest the presence of high-energy transients, such as those in pitting corrosion. Mesokurtic distributions (≈3) suggest uniform corrosion, and platykurtic values (<3) are associated with passive behavior due to the lack of strong events [57,58,59,60]: see Equation (11).

(10) $$k=\frac{\frac{1}{N}\sum_{i=1}^{N}{\left(x_{i}-\bar{x}\right)}^{4}}{\sigma^{4}}$$

### 2.4. HMM Parameter Calculation

The hidden states are defined as the corrosion types. The system follows automatic identification of present states and will only consider the ones present in the signal. Having generated “*N*” quantity of segments, the first probability to be calculated is the initiation probability through a search iteration for the first detected states throughout the dataset: see Equation (11).

(11) $$\pi_{j}=\frac{x_{j}}{M},\;where\;\sum_{j=1}^{N}\pi_{j}=1$$

The transition probability (*T_ij_*) is obtained first, through an identification of the present transitions (state A to state B), counting how often they appear and obtaining the probability that they are present throughout the database. The result from this is normalized, and the sum of each row must be 1: see Equation (12).

(12) $$T_{i,j}=\frac{C_{i,j}}{\sum_{k=1}^{N}C_{i,k}},\;where\;\sum_{j=1}^{N}T_{i,j}=1,\;\;\forall i\in \left\{1,\;2,\;\ldots ,\;N\right\}$$

For the emission probability (*b_j_*), since we are not working with the traditional discrete observations and given our assumptions during the statistical calculations for Gaussian-like behavior, we assume that a probability distribution will also follow the same criteria for a Gaussian distribution in order to model the continuous observations for a *j* state, where *μ*_*j*_ represents the median and $\sigma_{j}^{2}$ is the variance [61,62]: see Equation (13).

(13) $$b_{j}\left(o_{t}\right)=\frac{1}{\sqrt{2\pi \sigma_{j}^{2}}}exp\left(-\frac{{\left(o_{t}-\mu_{j}\right)}^{2}}{2\sigma_{j}^{2}}\right)$$

## 3. Results and Discussion

The pH of the electrolytes and the Open Circuit Potential (OCP) were measured to provide context for interpreting the corrosion behavior observed in the subsequent results. pH measurements were performed at room temperature using a calibrated pH meter, following a 20-min stabilization period prior to electrochemical noise (EN) testing. OCP measurements were obtained by recording the potential of each system over a 30-min period under open-circuit conditions, following an initial 10-min stabilization phase (see Table 3).

In the corrosion kinetics analysis, only current values were considered in all calculations. The signals were processed in grouped sets, with ten signals corresponding to each electrolyte. The procedure involved loading each signal, segmenting it through the PELT algorithm, and subsequently classifying each segment according to its corresponding corrosion type. The states identified across the signals were used to construct the initial and transition probability matrices. Meanwhile, the data points within each segment were used to estimate the probability densities required for the emission matrix.

Beginning with Group 1—AA2055 in 5 wt.% H_2_SO_4_ solution, the complete segmentation of all signals resulted in a total of 60 segments, of which 49 segments were classified as general corrosion, with 8361 data points, 3 segments were classified as localized corrosion, with 1010 data points, and 8 segments were classified as mixed corrosion, with 669 data points.

**Figure 2:**
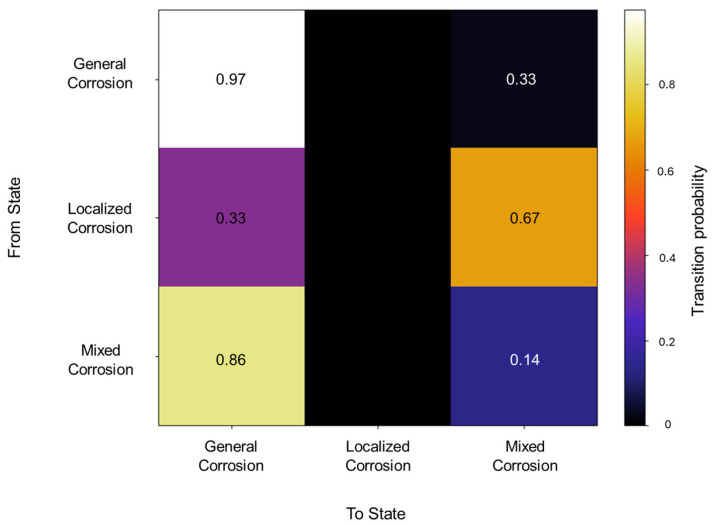
Transition probability matrix of Group 1—AA2055 exposed to 5 wt.% H_2_SO_4_ solution.

The initiation matrix states an equal probability of 0.3 for general and localized corrosion, with a slightly higher probability of 0.4 of initiating mixed corrosion across the database. A total of 50 transitions were detected across the database. The transition matrix in Figure 2 reflects that within general corrosion, there is a 0.97 probability of maintaining said state, with no transition to localized corrosion and a 0.03 probability of transitioning to mixed corrosion. The localized corrosion has a 0.33 likelihood of transitioning to general corrosion and a 0.66 probability of transitioning to mixed corrosion, with no persistence in its own state. Mixed corrosion presented a 0.85 probability of transitioning to general corrosion, with a likelihood of 0.14 of remaining in the same state. It is noteworthy that there are no transitions towards localized corrosion and that its appearance is strictly present within the same state or mixed corrosion.

**Figure 3:**
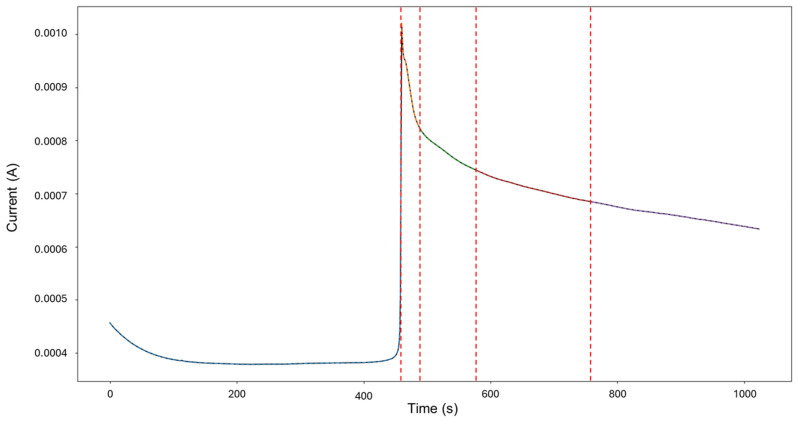
Segmentation of signals 1–7 of Group 1 AA2055 exposed to 5 wt.% H_2_SO_4_ solution.

The targeted signals “1–7” were selected for targeted HMM analysis. The calculations for traditional methods signify a skewness of 0.0523 and kurtosis of —1.462, indicating general corrosion. The segmentation, as seen in Figure 3, indicates an initial detection of localized corrosion with a total of 460 data points, followed by four segments of general corrosion.

Visually, the signal depicts low values of current, starting at 4.56 × 10^−4^ A and maintaining stability. After approximately 450 s, a sharp increase occurs, reaching 1.02 × 10^−3^ A, with no additional or accompanying transients. Following this, at around 463 s, the signal presents a slow decay trending towards stability. This phenomenon of a large single transient occurring mid-testing can be attributed to a distinct aggressive localized attack that undergoes passive action as quickly as it started.

**Figure 4:**
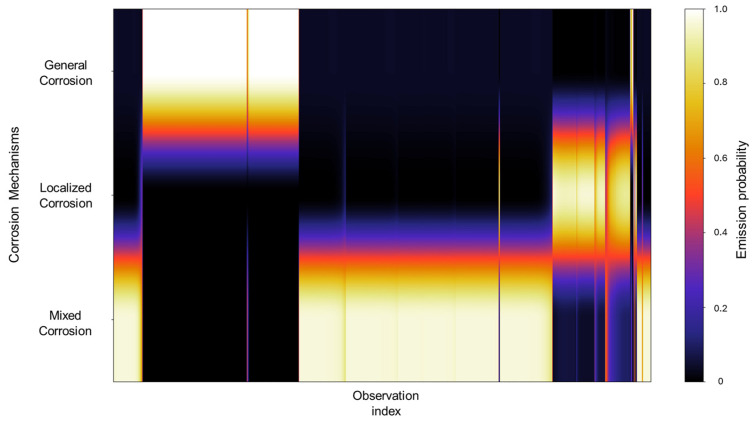
Emission probability map for Group 1 AA2055 exposed to 5 wt.% H_2_SO_4_ solution.

The emission probabilities show very distinctive values, with little to no overlap present between states, as shown in Figure 4. This indicates that the signals in the database show behavior that is distinctive enough, enabling the HMM to effectively differentiate between them. A large part of the concentration can be found in the mixed corrosion, with a gradient towards localized corrosion.

The emission probabilities represent the likelihood of observing specific EN current values for a particular corrosion mechanism. Given the large size of these emission matrices, direct numerical representation like the transition matrix is impractical, considering that emission matrices encompass all the data belonging to the database. To address this, a heatmap is employed to visually represent the distribution of emission probabilities across all observations. In this format, lighter colors indicate higher probability densities, suggesting a stronger association between the observation and a particular corrosion mechanism, whereas darker colors correspond to lower probabilities. A concentrated area generates a cluster of lighter tones along a specific corrosion mechanism row, signifying that a substantial portion of the signal observations are strongly associated with that mechanism. In contrast, regions that display gradients or spread across multiple mechanisms may reflect a transitional behavior, uncertainty in classification, or overlapping signal characteristics. Depending on the context, this may indicate the presence of mixed corrosion states, fluctuations between mechanisms, or potential misclassifications. In Figure 4, we can identify very distinctive values with little to no overlap present between states; this indicates that the signals in the database show behavior that is distinctive enough, enabling the HMM to effectively differentiate between them. A large part of the concentration can be found in the mixed corrosion with a gradient towards localized corrosion.

Before delving into the interpretation of HMM reconstruction, it is important to remember that it operates under a different analytical overview; instead of identifying discrete changepoints based on statistical shifts like PELT segmentation or generating global measures to assess overall variability, like in traditional calculations, it interprets the signal on the basis of probabilistic state transitions and learned patterns across time. As such, HMM is not limited to capturing only magnitude changes; it also employs the analysis of temporal behaviors and sequence structure. Consequently, the corrosion classifications produced by HMM may differ from those of PELT or traditional calculations, not as a contradiction but rather as a complementary perspective. While alignment across methods can occur, it should not be expected to be the norm.

**Figure 5:**
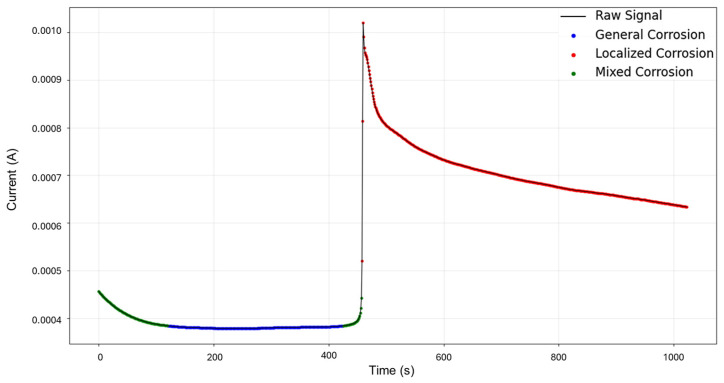
HMM reconstruction for signals 1–7 of Group 1—AA2055 exposed to 5 wt.% H_2_SO_4_ solution.

HMM reconstruction performed on signals “1–7”, as shown in Figure 5, shows very insightful behavior. An initial subtle decrease in current is classified as mixed corrosion. As the signal stabilizes, this region transitions into general corrosion. When the signal begins to rise again, it is once more identified as mixed corrosion. A subsequent and pronounced jump is then interpreted as localized corrosion, a classification that persists for the remainder of the signal. This pattern is coherent with physical corrosion processes: the early presence of mixed and general corrosion could indicate surface irregularities or the initiation of a pit. The transition to persistent localized corrosion suggests that the defect progressed and intensified over time. Furthermore, the eventual stabilization observed visually may indicate the onset of passivation; however, no formal classification pattern for passivation is currently implemented in the model.

The database of Group 2—AA2055 in 5 wt.% HCl solution—presents a behavior with high activity reflected by frequent transients. Additionally, most signals possess a striking resemblance to one another; thus, there is not much diversity behavior-wise, but there are plenty of meaningful transients.

The detected segments are comprised of 70 segments of 10,190 data points classified as general corrosion, with only a singular mixed corrosion event with 50 data points. The traditional calculations for the database signals also mostly signified general corrosion, with two signals indicating mixed corrosion. The initial state probability presented only general corrosion as the first probable event.

**Figure 6:**
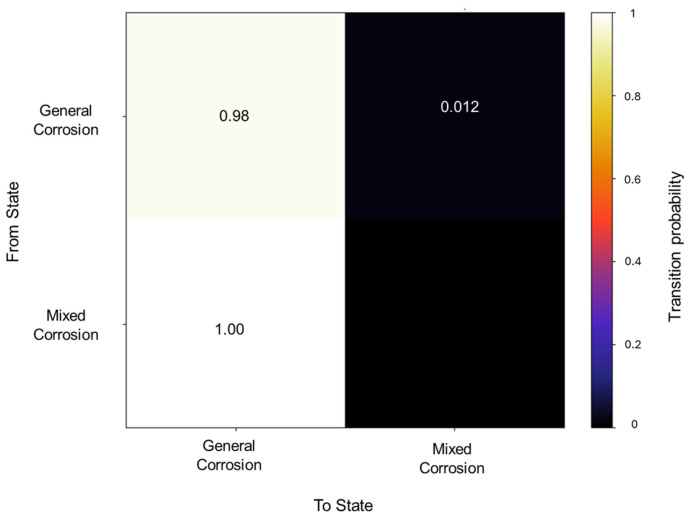
Transition probability matrix of Group 2—AA2055 exposed to 5 wt.% HCl solution.

The transition matrix shown in Figure 6 reveals that the general corrosion has an almost absolute probability of maintaining the same mechanism once it reaches a value of 0.98, while the transition from general corrosion towards mixed corrosion has a remaining value of 0.01. The transition probabilities in the mixed corrosion maintain an absolute value from mixed corrosion to general corrosion. This lack of more varied behavior is due to the dataset’s characteristics and behavior. The lack of variability among the signals will be reflected in the calculations.

**Figure 7:**
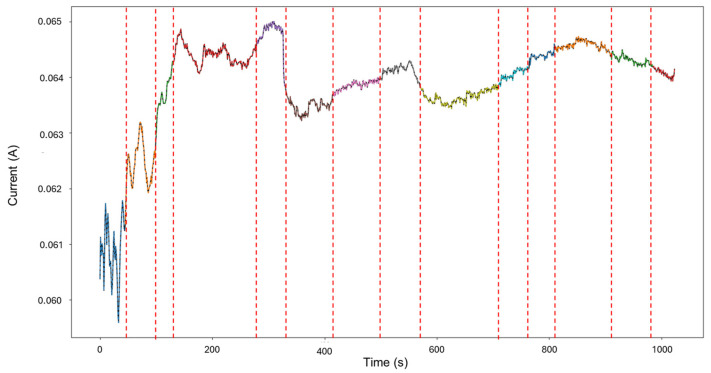
Segmentation of signals 2–6 of Group 2—AA2055 exposed to 5 wt.% HCl solution.

Signals 2–6 were selected for the analysis, as they presented an interesting behavior of mixed corrosion in the global calculations, with a skewness of −2.239 and a kurtosis value of 5.943. The segmentation of the signal observed in Figure 7 yielded 12 segments, of which the first 4 corresponded to general corrosion, and segment 5 was classified as mixed corrosion, while the rest of the segments were classified as general corrosion. Active signals can give the visual illusion that their presence must equate to localized attacks, but this is not necessarily the case. If the transients are found to be numerically balanced, the behavior is more consistent with general corrosion.

**Figure 8:**
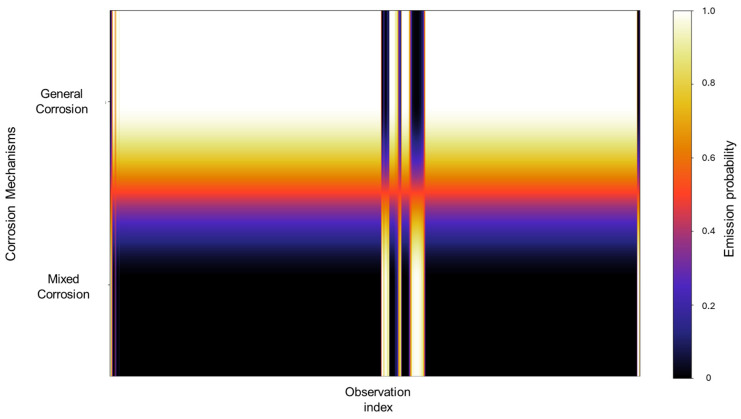
Emission probability map for Group 2—AA2055 exposed to 5 wt.% HCl solution.

In Figure 8, we can observe that the emission probability values present a strong concentration towards general corrosion, where most of the observations are clustered in this category, with a gradual decline towards mixed corrosion. Additionally, three narrow bands are associated with mixed corrosion. This distribution indicates that most of the data used to train the model are associated with general corrosion, with only a few distinct regions contributing to the classification of mixed corrosion. As a result, the model’s learning is heavily biased toward general corrosion behavior, which may affect its sensitivity to subtler forms of mixed or localized corrosion. However, this does not constitute a flaw in the model and is rather a consequence of the dataset’s characteristics and features.

**Figure 9:**
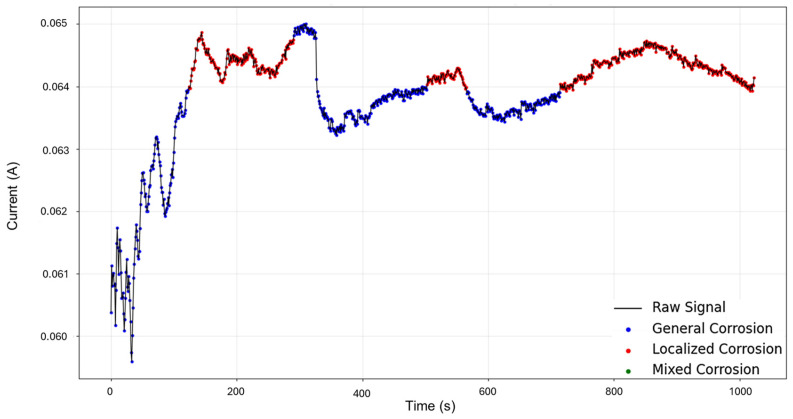
HMM reconstruction for signals 2–6 of Group 2—AA2055 exposed to 5 wt.% HCl solution.

In Figure 9, the signal reconstruction through HMM showcases the beginning of the signal as general corrosion and continues in this fashion until approximately 130 s, where it turns to mixed corrosion. The signal returns to a general corrosion state after 290 s, and up to around 500 s, the behavior mirrors that of the PELT segmentation. There is then a period of mixed corrosion from 503 to 565 s, followed by a general corrosion interval that is later disrupted by the presence of mixed corrosion at 715 s, which remains as such until the end of the signal.

Despite the database limitations in regard to the lack of detected corrosion states in the first stage, the HMM was successful in detecting meaningful areas that align with the expectations of the presence of mixed corrosion. The previously mentioned global results indicated mixed corrosion in a very particular way: the skewness value corresponded to general corrosion, while kurtosis was found within the range of localized corrosion, particularly intergranular SCC#1. This discrepancy can be better understood through HMM reconstruction, as we can now see where each of those components is located. This also underscores the reality that on signals such as these, there is room for speculation as to what exactly is occurring and when.

Group 3—AA2055 in 3.5 wt.% NaCl solution—is a very particular collection of signals. The EN responses of the samples under NaCl were almost identical in their entirety, with few notable differences in the signal’s signature, and unlike the previous group, this collection does not present pithy transients. The combined corrosion groups across all signals garnered 61 segments of general corrosion with 9861 data points, 2 segments of mixed corrosion with 309 data points, and a singular segment of localized corrosion with 70 data points. The general behavior of this database has very little variability.

**Figure 10:**
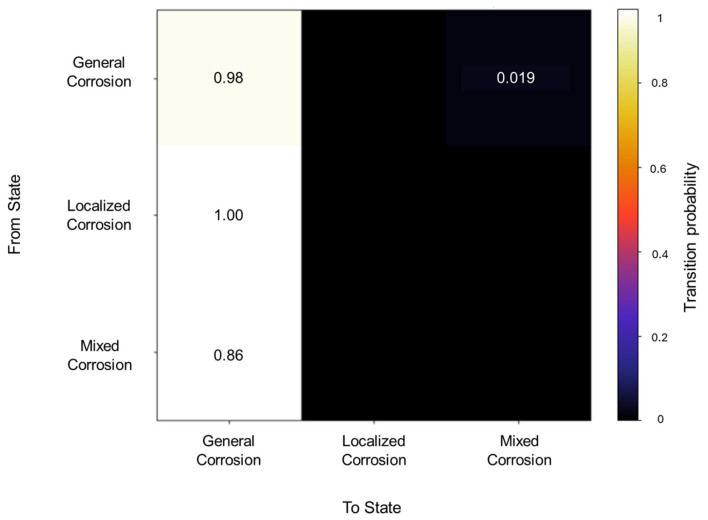
Transition probability matrix of Group 3—AA2055 exposed to 3.5 wt.% NaCl solution.

The initiation matrix showed a 0.8 probability of starting with general corrosion, with an equal probability of 0.1 for localized and mixed corrosion. A total of 56 transitions were detected across the database, of which the transition matrix in Figure 10 shows a higher probability (0.98) of general corrosion staying within the same corrosion type and a 0.019 probability of transitioning from general to mixed corrosion. With regards to localized and mixed corrosion, they appear to only occur within themselves. This could be attributed to a limited number of transitions detected for both cases. However, this reflects the reality of the signal’s behavior, thus limiting signals with low variability.

**Figure 11:**
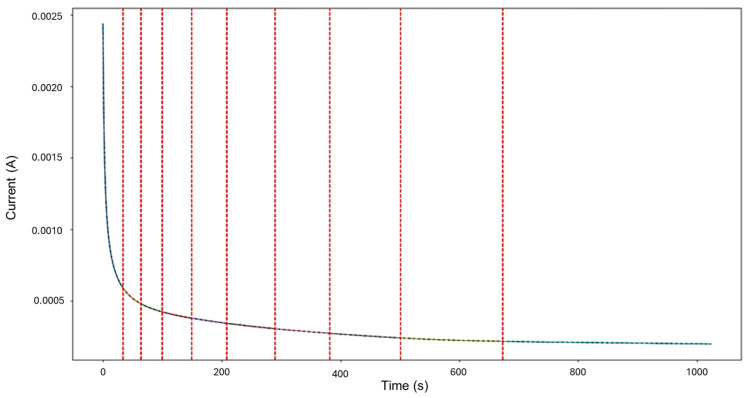
Segmentation of signals 3–4 of Group 3—AA2055 exposed to 3.5 wt.% NaCl solution.

Signals 3–4 were selected for targeted HMM reconstruction. The traditional calculations resulted in a skewness of 5.833 and kurtosis of 53.946; these values, particularly kurtosis, signify a strong deviation from normality and are consistent with localized corrosion, particularly intergranular stress corrosion cracking Type 2 (SCC2). The signal depicts a starting value of 2.44 × 10^−3^ A, followed by a drastic logarithmic-like decline until approximately 200 s, after which it begins to stabilize around a minimum value of 1.97 × 10^−4^ A. This behavior is often associated with passivation, as the system reaches a low steady state and does not show variability. The segmentation shown in Figure 11 detected the initial segment to be localized corrosion with a length of 35 data points, followed by nine segments categorized as general corrosion. This suggests that the most common and prevalent behavior is general corrosion, which is not reflected in the traditional calculation. The caveat in this disparity is that the traditional calculation suggests a localized attack akin to cracks, but visually, we see no high interval variability that could suggest this; what instead could be happening is that the large range of the cathodic drop greatly influences and skews the results. This effect is common when a single major event occurs early, followed by signal stabilization.

The emission probabilities derived from the database reveal a gradient-like distribution, concentrating mostly on general corrosion. This is gradually decreased in localized corrosion and then increases once again in mixed corrosion, although not to the extent of general corrosion. Notably, localized corrosion presents striking thin bands of concentration. This pattern signifies that, due to the relative similarity among signals and the overall low noise activity, there are fewer distinct features for the model to detect, which results in more diffuse classification boundaries.

**Figure 12:**
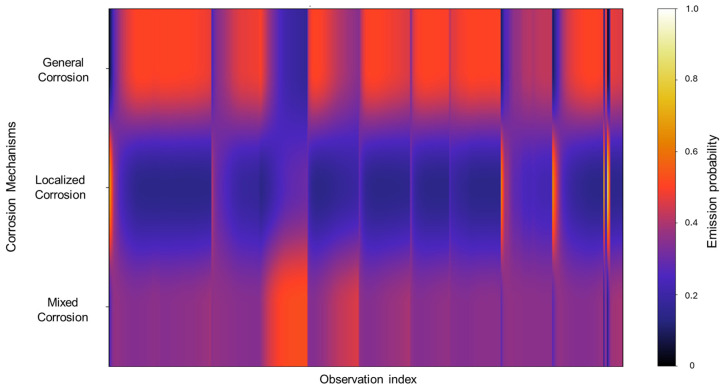
Emission probability map for Group 3—AA2055 exposed to 3.5 wt.% NaCl solution.

**Figure 13:**
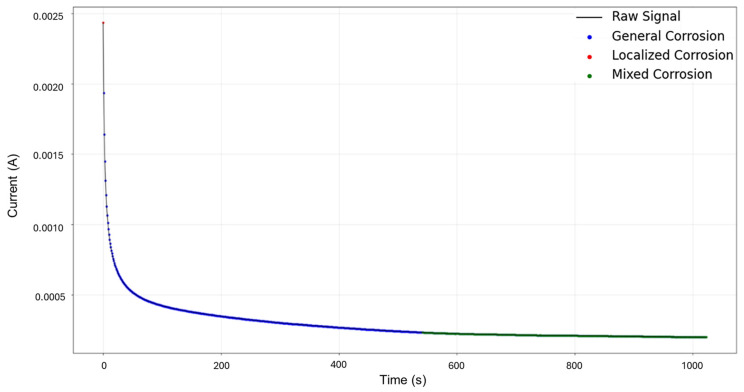
HMM reconstruction for signals 3–4 of Group 3—AA2055 exposed to 3.5 wt.% NaCl solution.

The results of HMM reconstruction show that the beginning of the signal is classified as general corrosion, with later events categorized as mixed corrosion. When comparing these findings with the segmentation results, some discrepancies become evident, which can be explained by the behavior observed in Figure 12. In this case, the limited variability and strong similarity within the database restrict the HMM’s ability to capture all the subtle nuances present in the signal. Nevertheless, it is clear that localized corrosion is not the dominant behavior and is scarcely present. This outcome demonstrates that the HMM is capable of moving beyond the imperfections introduced by traditional statistical methods (Figure 13). However, it also highlights that further fine-tuning is necessary to improve model accuracy, specifically by using a larger and more varied database. Recognizing the limitations of the proposed approach is crucial; feeding the model only signals with low or negligible variability limits its ability to accurately distinguish between different corrosion mechanisms. Variability within the dataset is essential for the successful application and reliability of this modeling strategy.

In the case of Group 4—AA2055 in Tap water MTY—the overall current values are much lower than previous groups. This is due to the electrolyte itself not being as aggressive as the previous media. The signal collection present in this group showcases large symmetrical transients with considerably different EN signatures overall. The most prevalent corrosion type, according to traditional calculations, is general corrosion, with a singular signal presenting mixed corrosion. This signifies that there is little to no diversity, as far as the corrosion process is concerned, and this must be taken into consideration when interpreting the results.

**Figure 14:**
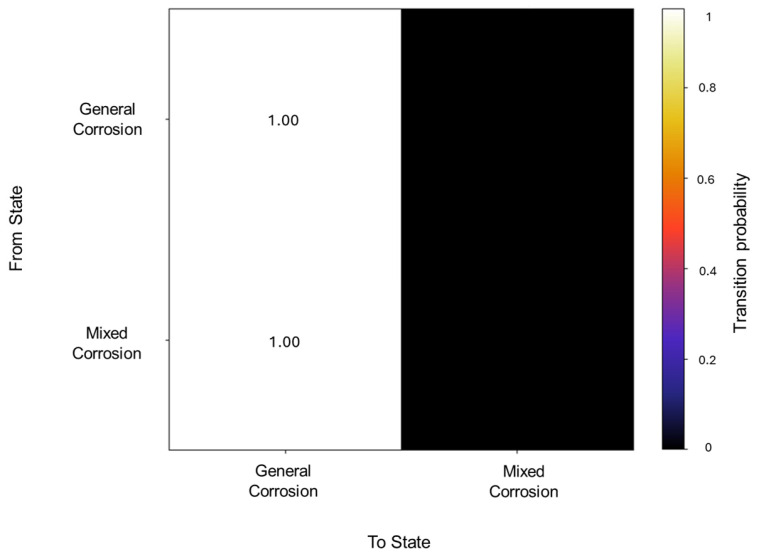
Transition probability matrix of Group 4—AA2055 exposed to Tap water MTY.

In the segmentation process, 79 segments were found to be general corrosion, with a total of 10,200 data points, and a segment was classified as mixed corrosion, with 40 data points. The initiation matrix presents a 0.9 probability of starting with general corrosion and a 0.1 probability of mixed corrosion. Focusing on the transition matrix in Figure 14, the results reflect the absolute probability of general corrosion maintaining its state, with the same case for mixed corrosion.

**Figure 15:**
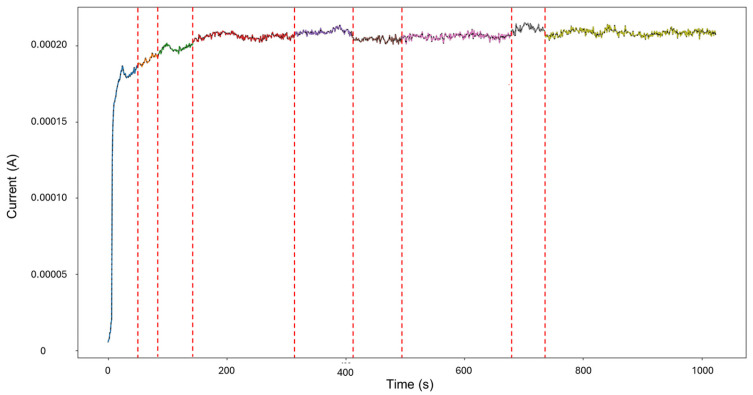
Segmentation of signals 4–5 of Group 4—AA2055 exposed to Tap water MTY.

For HMM reconstruction, the signals “4–5” were selected, as this presented the only case of mixed corrosion in the database. The PELT segmentation results are shown in Figure 15 and present nine segments, of which all were classified as general corrosion. The traditional calculations indicated a skewness of —9.00763599, which is aligned with general corrosion, and a kurtosis value of 93.17755502, which would suggest a heavily localized attack. This discrepancy is similar to the one observed in the signals “3–4”, where a large singular jump can heavily influence the global results and lead to confounding interpretations. The signal commences with a value of 5.52 × 10^−6^ A at approximately 8 s, and there is a sharp increase in current at 25 s, reaching 1.87 × 10^−4^ A. The signal continues with slight increases and maintains stability at around 2.15 × 10^−4^ A, with frequent low-amplitude transients.

**Figure 16:**
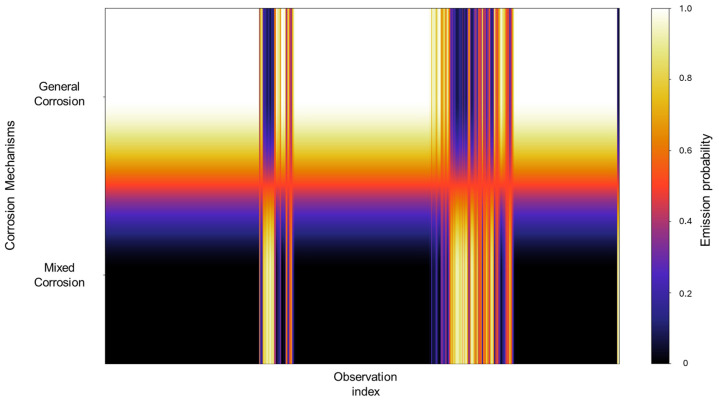
Emission probability map for Group 4—AA2055 exposed to Tap water MTY.

The values observed in the emission probability matrix in Figure 16 reflect the dominant presence of general corrosion, with mixed corrosion showing clearly discernible values. Although not as frequent as general corrosion, the distinct separation between the two indicates that, despite the overall similarity within the database, the signals retain enough unique characteristics to allow the model to meaningfully reconstruct the signal behavior.

**Figure 17:**
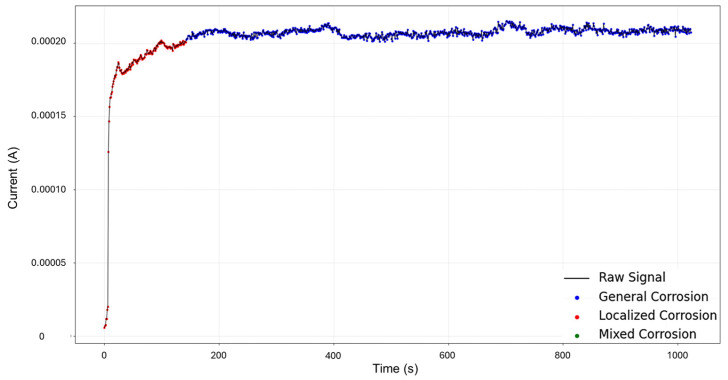
HMM reconstruction of signals 4–5 of Group 4—AA2055 in Tap water MTY.

The result of the reconstruction showcases the initial portion of the signal as mixed corrosion. After approximately 150 s, the signal transitions to general corrosion, which is maintained throughout the remainder of the signal (see Figure 17). The behavior of the signal aligns with reality: the early anodic jump exhibits the highest level of fluctuation, consistent with mixed corrosion. In contrast, the subsequent general corrosion phase is characterized by mild and stable transients, supporting the classification provided by the HMM.

The database of group 5—AA2055 in Tap water GP—presents varying signatures, with some of the signals presenting more stable transients, while others appear to be quite noisy. It is important to note that Gomez Palacio recognized a zone of chronic hydroarsenicism; in response, the government implemented intensive water treatment processes, including reverse osmosis, oxidation filtration, and activated alumina absorption [63]. However, despite these measures, the water has still been deemed unsafe for consumption, as arsenic and fluoride levels remain elevated beyond recommended limits. These persistent contaminants, as well as the aggressive treatment protocols, likely contribute to the variability observed in the electrochemical noise signals, as they can influence the electrochemical interface differently across samples, resulting in heterogeneous corrosion behaviors.

**Figure 18:**
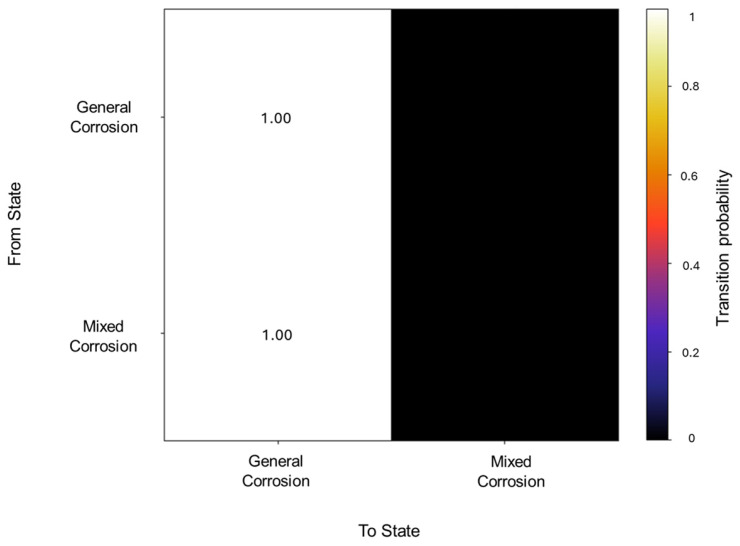
Transition probability matrix of Group 5—AA2055 exposed to Tap water GP.

The traditional calculations reflected this diversity, with six signals presenting general corrosion and four signals presenting mixed corrosion. The overall segments obtained from the signals are comprised of 69 segments of general corrosion, with 9895 data points, and 3 segments for mixed corrosion, with 345 data points. The initial state probabilities reflected a higher likelihood of presenting general corrosion as the first state, with a 0.7 probability, while mixed corrosion presented a 0.3 probability (see Figure 18).

**Figure 19:**
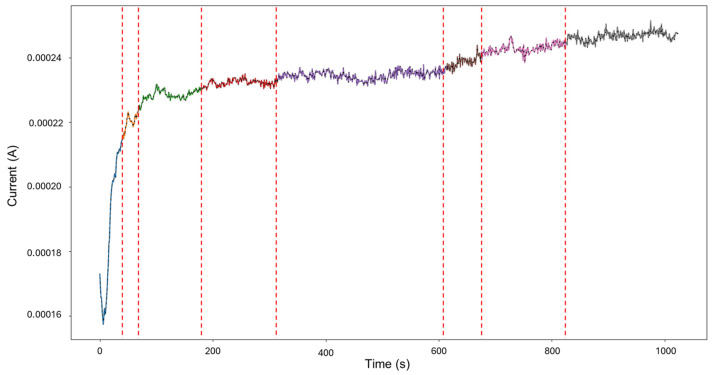
Segmentation of signals 5–10 of Group 5—AA2055 exposed to Tap water GP.

A total of 62 transitions were detected, from which the transition probabilities presented absolute values for general corrosion within the same state, as well as mixed corrosion (see Figure 19). This signifies that the states occur in an isolated manner and do not deviate from the state once they have entered it.

The signals chosen for HMM reconstruction were “5–10”, with mixed corrosion as the most prevalent behavior according to traditional calculations. The results from said operation offered a skewness of −3.291 in accordance with general corrosion and a kurtosis of 15.806, which falls within the localized attack of transgranular SCC#2. The disparity in the results indicates that the signal presents varying behaviors that cannot be isolated by the traditional methodology.

The recordings initiated at a current value of 1.73 × 10^−4^ A, and at approximately 6 s, the values dropped, potentially signifying an early stabilization or the brief onset of passivation. Shortly after around 30 s, the signal abruptly increased to 2.10 × 10^−4^ A. From this point forward, the signal presented active transients, with some regions exhibiting relatively symmetrical transients, while others showed more pronounced asymmetry. This variation supports the presence of mixed corrosion. The PELT segmentation identified eight segments within the signal, and the initial section was classified as mixed corrosion, likely due to the irregular and pronounced activity early on, while the remaining segments were categorized as general corrosion, reflecting a more stable and uniform pattern.

**Figure 20:**
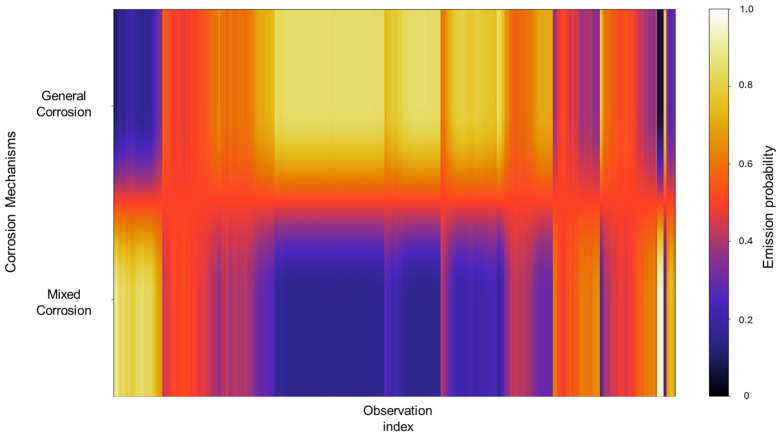
Emission map for Group 5—AA2055 exposed to Tap water GP.

The emission probability calculations behave similarly to those observed in Group 3. Rather than exhibiting completely distinct zones for each corrosion type, the results show a gradient of probabilities between general and mixed corrosion. At the beginning of the observations, the distribution leans more toward mixed corrosion; however, as the signal progresses, general corrosion begins to dominate gradually. This back-and-forth dynamic continues, with a consistent proclivity toward general corrosion. Such gradient-like results suggest that the system does not experience abrupt transitions between corrosion mechanisms but rather fluctuates within a spectrum of behaviors (Figure 20).

**Figure 21:**
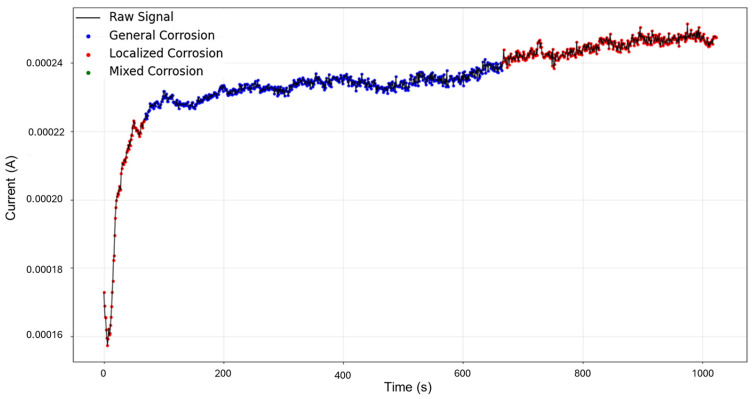
HMM reconstruction of signals 5–10 of Group 5—AA2055 exposed to Tap water GP.

The signal reconstruction through HMM exhibits both general and mixed corrosion, as expected (Figure 21). The initial segment of the signal aligns with the PELT segmentation, indicating mixed corrosion. This is followed by a period of relative stability characterized by uniform transients, corresponding to general corrosion. Around 650 s, the signal begins to exhibit noisier transients with varying amplitudes, signaling a shift back to mixed corrosion. This suggests the coexistence or alternating influence of general and localized corrosion mechanisms as the system evolves over time.

PELT segmentation demonstrated successful identification of key changepoints, establishing itself to be an excellent tool for the interpretation of EN signals. The selection of penalty value through an iterative process for best fitting allowed the algorithm to adapt to the signals in a dynamic manner that ensures the behavior of the signal is accounted for, rather than choosing an arbitrary value. The application of this technique represents a significant improvement and solid alternative to the often-used sliding window technique [64,65], which requires a manual selection of window length and step size, values that are often arbitrarily defined and may not align with the underlying structure of all signals. Instead, PELT makes it possible to capture the transition more accurately, as events of interest may not be uniformly distributed across time.

Group 1—AA2055 in 5 wt.% H_2_SO_4_ solution (high variability in behavior, few transients, and a good amount of diversity of corrosion processes)—presented the least amount of total segmentation, with 60 generated segments, in which the detection of generalized, localized, and mixed corrosion was found. This database presented both quick exponential anodic rises that approach stability at higher current values and sharp sudden increases with a slow decay, as seen in Figure 3.

Group 2—AA2055 in 5 wt.% HCl solution (high variability in behavior, many transients, and low diversity in corrosion processes)—accumulated a total of 71 segments, of which just 1 belonged to mixed corrosion, with the rest belonging to general corrosion. This dataset presented both high variability of behavior in the signatures and noisy transients, and it presented a successful reconstruction of the signal.

Group 3—AA2055 in 3.5 wt.% NaCl solution (low variability in behavior, low transients, and moderate diversity of corrosion processes)—possesses a total of 64 segments, with 2 segments of mixed corrosion, a singular event of localized corrosion, and the rest pertaining to general corrosion. This dataset presented very similar signatures with constant decays without reaching full stability, and some presented plateaus, followed by a continued cathodic drop. HMM reconstruction presented some discrepancies, likely induced by the lack of distinctive behaviors of the signals in the database.

Group 4—AA2055 in Tap water MTY solution (high variability in behavior, high variants of transients, and low diversity of corrosion processes)—presented the greatest number of segments, with a total of 80, in which all but 1 segment was classified as mixed corrosion. This dataset presents the most active transients with somewhat similar signatures. HMM reconstruction was successful.

Group 5—AA2055 in Tap water GP solution (high variability in behavior, many transients, and low diversity of corrosion processes)— consisted of 72 total segments. This dataset presented varying signatures with moderate to large transients. This showed a large amount of activity and different responses. HMM reconstruction was successful.

From these results, we can affirm that the number of segments produced did not have an effect on HMM reconstruction, given that the lowest segmentation, present in Group 1 (AA2055 in 5 wt.% H_2_SO_4_ solution) and consisting of 60 segments, and the highest one, belonging to Group 4 (AA2055 in Tap water MTY), both presented satisfactory HMM reconstructions that align with the expected corrosion behavior. Group 3—AA2055 in 3.5 wt.% NaCl solution—presented the least favorable conditions for HMM reconstruction. Although reconstruction was still successful, the emission matrix presented significant overlap across corrosion types, likely due to a lack of distinctive emission patterns, given that the dataset presented low variability; ergo, the signals displayed highly similar behaviors that led to the overlap, resulting in a broad spread of, rather than concentrated, emission probabilities, thus resulting in lower distinguishing capabilities compared to that of the other groups with more clear-cut state labels.

Nevertheless, the system showed a preference for general corrosion, showing that despite the ambiguity present, dominant patterns could still be identified.

Another interesting emission distribution was that of Group 5 (AA2055 in Tap water GP solution), which also presented some degree of overlap within the corrosion types. However, this was not nearly as severe as the previous group, showing clear and distinct zones of emission distribution, attributable to their respective corrosion types. This group presented a successful reconstruction with no discrepancies.

The challenges regarding Group 3 (AA2055 in 3.5 wt.% NaCl solution) highlight the importance of the emission matrix calculations for the HMM system, and that ambiguity can lead to misclassification. Sharing this perspective, the work of Volant et al. [66] demonstrates that even minor inaccuracies in parameter estimation can lead to unreliable results, thus highlighting the need to focus on developing more flexible algorithms to improve the estimation of emission distributions. Srivastava et al. [67] developed an improved classification of the HMM by modifying the emission probabilities with negative training sequences to adjust emission probabilities and minimize the influence of non-discriminative signals to improve the model’s ability to differentiate between closely related sub-families. Ahola et al. [68] focused on generating a methodology for efficient emission probability estimation as a way to improve the accuracy of the HMM’s profile by means of the “EEP” method, which differentiates between effective and ineffective residues at each alignment position of the estimation calculations in order to reduce the number of free emission parameters and thus enhance specificity. The commonality we can perceive here is the constant search to improve the emission calculations and the accuracy to obtain better HMM results, regardless of the HMM being employed, given that emissions have a rather big influence on the HMM process, as we have been able to discern here.

An interesting angle to consider for future implementations is that the segments generated did not yield perfectly balanced distributions of corrosion types. This outcome was expected, as the goal was to capture the natural and unmanipulated behavior of the signals. As such, some datasets featured only certain types of corrosion, with segments varying significantly in size. While this approach preserves the authenticity of electrochemical behavior, it also poses challenges for model training and evaluation. Expanding the analysis to larger databases could help gather more representative data across all corrosion types, potentially enabling more balanced and robust HMM reconstructions. However, further research is needed to assess whether such expansion would lead to clearer improvements in classification performance or simply reflect the inherent variability of corrosion processes.

The limitations present on the application of HMM stem primarily from the Markovian assumption, which states that each state transition depends solely on the previous state, neglecting long-range dependencies or memory effects that may be present in electrochemical processes. Additionally, this technique relies on Gaussian emission distributions, which is the same limitation that bounds traditional statistical approaches to corrosion analysis. Moreover, uneven class distributions, limited dataset sizes, and low signal variability can hinder the model’s ability to generalize, learn distinctive patterns, and produce meaningful reconstructions. 

When addressing the instances of differences between the statistical classification of segments and HMM reconstruction, it is important to note that divergence in the results is not a reflection of contradiction but rather evidence of their different analytical frameworks. Statistical analysis generates isolated results based on local descriptors [57,58,59], while HMM considers temporal sequence and transition likelihoods between states [40,41,42,43,44,45,46,47], thus offering a more dynamic response by evaluating the data in the context of the entire signal sequence and learned state behavior. Therefore, such mismatches do not necessarily indicate a flaw but demonstrate the strength and limitations of each method; preference for one classification over the other will depend on the goal of the analysis and the quality of the database. If one seeks to characterize instantaneous signal traits, statistical methodology will be sufficient; however, if the goal is to understand the corrosion process and its progression, HMM will provide a more robust interpretation, provided that the emission probabilities, along with initiation and transition parameters, are well-tuned.

## 4. Conclusions

The aim of the present study was to successfully apply the HMM technique to EN signals of AA2025 exposed to various corrosive environments to improve the reliability and depth of corrosion type classification compared to traditional methods. A secondary aim was the integration of the PELT algorithm to segment the signals to obtain the necessary classificatory features required for the HMM parameters and subsequent signal reconstruction. Both objectives were successfully accomplished, and their introduction to EN studies contributes to corrosion analysis by innovating the traditional methodologies and introducing new computationally robust techniques. Based on the values obtained from HMM reconstruction, the following conclusions can be drawn:

- Group 1—AA2055 in 5 wt.% H_2_SO_4_solution: Traditional results classified the signal as general corrosion, although visual inspection and PELT segmentation revealed both localized and general corrosion patterns. HMM reconstruction delivered an initial slow decay of mixed corrosion, followed by a general corrosion phase, culminating in a high anodic spike, indicating localized corrosion. This result aligned better with the signal behavior than the traditional classification.
- Group 2—AA2055 in 5 wt.% HCl solution: Traditional analysis identified mixed corrosion, which was corroborated by the statistical classification of segments, showing the presence of both general and mixed corrosion. HMM reconstruction provided a clearer differentiation between these two states, demonstrating its capability to distinguish complex signal transitions and showcase the behavior of the signal beyond the singular traditional result.
- Group 3—AA2055 in 3.5 wt.% NaCl solution: Traditional results suggested intergranular stress corrosion cracking (IGSCC), but visual analysis contradicted this, as no sharp transient was present that could be attributed to a crack. Segmentation classification indicated a mix of localized and general corrosion, and HMM results reflected general corrosion, followed by mixed corrosion. However, due to significant overlap in emission probabilities, state resolution was limited, highlighting the importance of emission matrix optimization for reliable reconstruction.
- Group 4—AA2055 in Tap water MTY solution: While traditional classification indicated mixed corrosion, segment classification showed consistent general corrosion throughout the segments. HMM reconstruction demonstrated a mixed corrosion state during the anodic-transient-rich phase, followed by general corrosion, which is indicative of HMM’s strength in resolving transitional dynamics within the signal, as well as its ability to determine what occurs in the mixed corrosion category in the traditional classification.
- Group 5—AA2055 in Tap water GP solution: Traditional and segment-based classifications both pointed to a combination of mixed and general corrosion. HMM reconstruction confirmed this, identifying two broad regions of mixed corrosion, with a stable central period classified as general corrosion.

This study on EN signals using HMM has proven valuable for decoding complex corrosion behavior over time, showing particularly interesting patterns in the cases where the traditional methodology does not account for mixed corrosion. The most accurate results are proven to occur when emission parameters are carefully estimated and supported by datasets with enriching distinct behaviors. These findings suggest that HMM-based approaches can yield more insightful interpretations than static traditional methods. Further research is necessary to reinforce and generalize these findings by expanding datasets with a broader range of segments and corrosive events. Moreover, incorporating additional electrochemical phenomena, such as passivation events, into the classification schema could increase the interpretive power of the model.

## Figures and Tables

**Table 1:** Chemical composition of AA2055 alloy (wt.%).

Aluminum Alloy	Elements							
	Li	Cu	Mg	Ag	Zr	Mn	Zn	Al
2055	1.15 ± 0.05	3.7 ± 0.18	0.4 ± 0.02	0.4 ± 0.02	0.11 ± 0.05	0.3 ± 0.01	0.5 ± 0.02	Balance

**Table 2:** Kurtosis and skewness interval values.

Corrosion Type	Potential		Current	
	Skewness	Kurtosis	Skewness	Kurtosis
General	<±1	<3	<±1	<3
Localized	<−2	≫3	>±1	≫3
Transgranular SCC	+4	20	−4	20
Intergranular SCC * #1	−6.6	18–114	1.5–3.2	6.4–15.6
Intergranular SCC * #2	−2 a−6	5–45	3–6	10–60

* SCC—stress corrosion cracking.

**Table 3:** Electrolyte characteristics.

ID. Designation Group	Electrolyte	pH	E_OCP_ (V)
1	H_2_SO_4_	0.07	−0.603
2	HCl	0.79	−0.772
3	NaCl	7.47	−0.850
4	Tap water MTY	8.23	−0.716
5	Tap water GP	8.34	−0.760

## Data Availability

The data presented in this study are available upon request from the corresponding author.
